# Thermally Tunable Orbital Angular Momentum Mode Generator Based on Dual-Core Photonic Crystal Fibers

**DOI:** 10.3390/nano11123256

**Published:** 2021-11-30

**Authors:** Lianzhen Zhang, Xuedian Zhang, Xuejing Liu, Jun Zhou, Na Yang, Jia Du, Xin Ding

**Affiliations:** Key Laboratory of Optical Technology and Instrument for Medicine, Ministry of Education, University of Shanghai for Science and Technology, Shanghai 200093, China; zlz16678188128@163.com (L.Z.); liuxuejing@usst.edu.cn (X.L.); jz361@outlook.com (J.Z.); 191550058@st.usst.edu.cn (N.Y.); 191380029@st.usst.edu.cn (J.D.); 201440055@st.usst.edu.cn (X.D.)

**Keywords:** thermally tunable, orbital angular momentum mode, generator, dual-core photonic crystal fiber

## Abstract

The combination of mode division multiplexing (MDM) based on orbital angular momentum (OAM) modes with wavelength division multiplexing (WDM) has attracted considerable attention due to its ability to increase optical transmission capacity. However, the switching of the multi-wavelength and multi-order OAM mode in an all-fiber structure has always been a challenge. As a solution, a thermally tunable dual-core photonic crystal fiber (DC-PCF) is proposed to achieve multi-order and multi-wavelength switching of the OAM mode. The results show that the OAM mode with topological charge m = ±1 can be excited with the linear polarization fundamental mode (LPFM) and circular polarization fundamental mode (CPFM). In addition, the device can effectively excite a high-purity ±1st order OAM mode with wavelengths ranging from 1520 to 1575 nm by thermal tuning. The purity of the mode is in excess of 99%, and the energy conversion efficiency (ECE) is above 95%. The proposed design is expected to be applied in all-fiber communication systems combined with MDM and WDM.

## 1. Introduction

In 1992, Allen first discovered the orbital angular momentum (OAM) of light [1], and then a large number of applied studies on OAM continued to deepen and expand our understanding of this topic [2,3,4,5]. Previous research showed that the application of OAM in optical fiber communication had great potential [6,7,8,9]. In 1998, Alexeyev discovered that OAM modes can be transmitted in optical fibers [10]. In 2013, Bozinovic was the first to verify that OAM mode multiplexing can be used for information transmission [11]. This provides a new solution for solving the capacity shortage crisis in optical fiber communication systems. The use of OAM mode multiplexing technology continues to enhance the capacity of optical fiber communication, and the transmission distance continues to increase. The communication distance has reached 100 km without optical amplification and the communication capacity has increased to 256 (Tbit/s)·km [12].

In principle, the OAM mode based on optical fiber can be multiplexed mainly due to its two characteristics: the number of topological charges of OAM can be infinite, and the OAM mode of different topological charges is orthogonal [13,14]. In addition to using OAM modes of different orders for multiplexing, to make full use of the OAM mode to transmit information, it is also possible to combine OAM mode division multiplexing (MDM) technology and wavelength division multiplexing (WDM) technology to increase communication capacity [15,16,17,18]. In a combined MDM and WDM communication system, the order of the OAM mode is switched through the spatial light modulator, and then it is coupled into the optical fiber through a precise coupling system. When these devices are combined with an optical fiber communication system, coupling loss and accurate optical alignment are factors that cannot be ignored. In contrast, if the all-fiber OAM mode generator can be used in this system to switch the OAM mode, the complexity and cost of system integration can be greatly reduced [19,20]. In order to cope with the demands, it is urgent to design an all-fiber OAM mode generator with adjustable OAM order and a wide wavelength range in a single structure.

To date, the optical fiber for OAM mode transmission mainly includes ring core fiber [12,21,22], and ring core photonic crystal fiber (PCF) [23,24]. In order to improve the coupling efficiency and reduce the melting difficulty, it is necessary to design the output structure of the all-fiber OAM mode generator as a ring. A review of the previous research works shows that OAM mode generators based on PCF are all realized by special design or microstructure processing of photonic crystal fibers [25,26,27,28,29]. Fu et al. used cascaded spiral PCF to generate the OAM mode, which is a relatively complex production process with a 35 nm working bandwidth [25]. Takeshi et al. converted the high-order mode to OAM mode based on the spiral PCF with high birefringence, instead of using the fundamental mode to excite the OAM mode [26]. Cui et al. verified the high-order OAM mode generation using coaxial two core PCF. The intermediate core is composed of two single-mode cores and is twisted. This structure is very difficult to implement, and this method can only excite OAM mode at a certain wavelength [27]. Seghilani et al. introduces a cross section of spiral effective refractive index distribution into PCF to excite the OAM mode. This structure needs to produce different orders by designing different distribution interfaces, and its purity decreases with the increase in the OAM mode order [28]. Fu et al. experimentally proved that by using torsional PCF, the OAM mode can be generated in its cladding [29]. After comparing the methods, it can be concluded that most of them cannot efficiently generate the OAM mode with high purity at each wavelength in its working bandwidth. In addition, the flexible filling ability of PCF is rarely used to achieve this function. In recent years, the continuous development of micro-processing technology has laid the foundation for the selective filling of PCF with liquid [30,31,32].

In this paper, we propose an OAM mode generator based on asymmetric dual-core PCF (DC-PCF). The DC-PCF is composed of a single-mode fiber core (SMFC) and a few-mode fiber core (FMFC), and six air holes around the SMFC are infiltrated with temperature-sensitive refractive index matching solution (TS-RIMS). The results indicate that the input of linear polarization fundamental mode (LPFM) or circular polarization fundamental mode (CPFM) has little effect on the coupling efficiency and purity, although the LPFM input requires a longer coupling area. Moreover, the device can efficiently generate high-purity OAM mode in a wide wavelength range. The structure proposed in this paper is flexible and controllable, and it provides a novel design for a multi-order adjustable and wide-wavelength all-fiber OAM mode generator.

## 2. Theoretical Analysis

In optical fiber, the OAM mode can be generated by the superposition of the odd and -even modes of the eigenmodes with π/2 phase difference, and the equation is as follows [3,33]:

(1) $$\begin{array}{l} {\mathrm{OAM}}_{\pm l}^{\pm }={\mathrm{HE}}_{l+1,m}^{\mathrm{even}}\pm i{\mathrm{HE}}_{l+1,m}^{\mathrm{odd}}\\ {\mathrm{OAM}}_{\pm l}^{\mp }={\mathrm{EH}}_{l-1,m}^{\mathrm{even}}\mp i{\mathrm{EH}}_{l-1,m}^{\mathrm{odd}} \end{array}$$

The superscript sign in ${\mathrm{OAM}}_{\pm l}^{\pm }$ or ${\mathrm{OAM}}_{\pm l}^{\mp }$ indicates the circular polarization direction of the vortex light, and the subscript sign indicates the rotation direction of the wavefront. l represents the number of topological charges of OAM. It can be seen from Equation (1) that the OAM mode synthesized by the HE mode has the same rotation direction as the polarization state, and the OAM mode synthesized by the EH mode has the opposite rotation direction as the polarization state.

Combining coupled-mode theory and super-mode theory [34], it can be concluded that the overall mode field of the DC-PCF can be expressed as Equation (2):

(2) $$\hat{E}\left(r\right)={\hat{E}}_{\mathrm{even}}\left(r\right)+{\hat{E}}_{\mathrm{odd}}\left(r\right)$$

where ${\hat{E}}_{\mathrm{even}}\left(r\right)={\hat{\varepsilon }}_{1}\left(x,y\right)e^{i\beta_{1}z}+{\hat{\varepsilon }}_{2}\left(x,y\right)e^{i\beta_{2}z}$ and ${\hat{E}}_{\mathrm{odd}}\left(r\right)={\hat{\varepsilon }}_{3}\left(x,y\right)e^{i\beta_{3}z}+{\hat{\varepsilon }}_{4}\left(x,y\right)e^{i\beta_{4}z}$, ${\hat{\varepsilon }}_{1}\left(x,y\right)$, ${\hat{\varepsilon }}_{2}\left(x,y\right)$, ${\hat{\varepsilon }}_{3}\left(x,y\right)$ and ${\hat{\varepsilon }}_{4}\left(x,y\right)$ are the field solutions of the supermodes generated when mode resonances occur in the two cores, $\beta_{1}$, $\beta_{2}$, $\beta_{3}$ and $\beta_{4}$ are the propagation constants of the four supermodes.

It can be seen from Equation (2) that the overall mode field of the DC-PCF can be expressed as the superposition of two pairs of supermodes. When CPFM is coupled into the SMFC, that is, z=0, the overall light field can be expressed as Equation (3).

(3) $$\hat{E}\left(r\right)={\mathrm{HE}}_{11}^{\mathrm{even}}\pm i{\mathrm{HE}}_{11}^{\mathrm{odd}}$$

According to the coupled-mode theory, the maximum energy conversion occurs between HE_11_ and HE_mn_ when the coupling distance is $L=\pi /\left(\beta_{1}-\beta_{2}\right)$, and all the energy can be transferred when the two fiber cores satisfy the phase matching condition. When the transmission distance *z* is an odd multiple of *L* and the phase matching condition is met, the energy of the fundamental mode will be fully coupled to the higher-order mode.

The overall mode field of the DC-PCF changes with the increase in *z*. When the transmission distance *L* satisfies Equation (4), where $k_{1}$, $k_{2}$, $k_{3}$ and $k_{4}$ are any positive integers, the overall mode of the fiber under the condition of phase matching can be expressed as Equation (5), and OAM mode can be activated at this length.

(4) $$L=\frac{2k_{1}\pi }{\beta_{1}}=\frac{\left(2k_{2}+1\right)\pi }{\beta_{2}}=\frac{2k_{3}\pi }{\beta_{3}}=\frac{\left(2k_{4}+1\right)\pi }{\beta_{4}}$$

(5) $$\hat{E}\left(r\right)={\mathrm{HE}}_{mn}^{\mathrm{even}}\pm i{\mathrm{HE}}_{mn}^{\mathrm{odd}}$$

When LPFM is coupled into the SMFC, that is, z=0, the overall mode field can be expressed as Equation (6).

(6) $$\hat{E}\left(r\right)={\mathrm{HE}}_{11}^{\mathrm{even}}\pm {\mathrm{HE}}_{11}^{\mathrm{odd}}$$

When the transmission distance *L* satisfies Equation (7), where, $q_{1}$, $q_{2}$, $q_{3}$ and $q_{4}$ are any positive integers, the overall mode field of the fiber under the phase matching condition can be expressed as Equation (8), and OAM mode can be activated.

(7) $$L=\frac{2q_{1}\pi }{\beta_{1}}=\frac{\left(2q_{2}+1\right)\pi }{\beta_{2}}=\frac{\left(2q_{3}+1/2\right)\pi }{\beta_{3}}=\frac{\left(2q_{4}+3/2\right)\pi }{\beta_{4}}$$

(8) $$\hat{E}\left(r\right)={\mathrm{HE}}_{mn}^{\mathrm{even}}\pm i{\mathrm{HE}}_{mn}^{\mathrm{odd}}$$

As analyzed above, by combining the theory of coupled modes and supermodes, it can be seen that the OAM mode can be generated when the coupling length meets Equation (4) with CPFM input; when LPFM is injected, the OAM mode can be generated when the coupling distance meets Equation (7). In the literature [35] this is called the “phase shift coupling condition” (PSCC).

## 3. Structure Design of OAM Mode Generator

**Figure 1:**
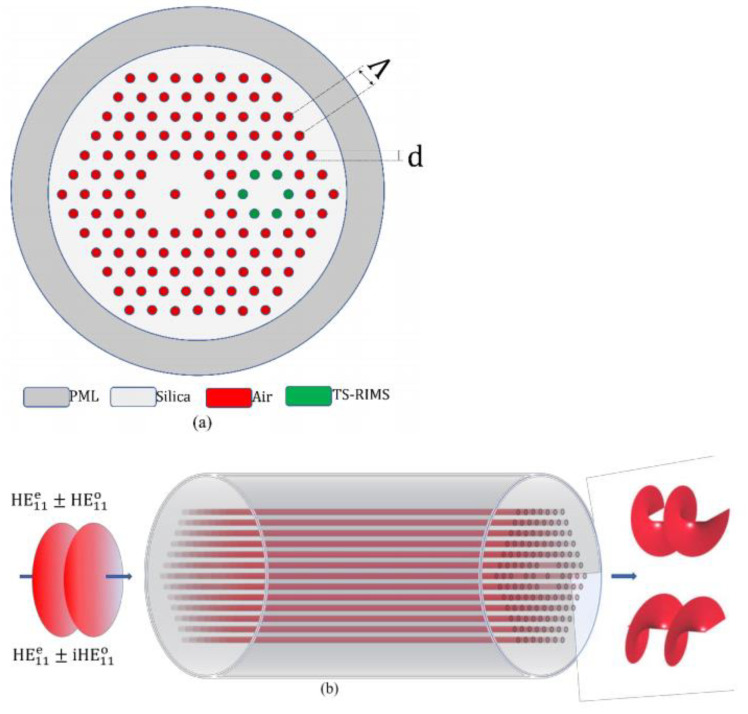
(**a**) Cross-section of the proposed DC-PCF OAM mode generator. (**b**) Working principle and structure diagram of DC-PCF OAM mode generator. The input light can be LPFM or CPFM. The ±1st order OAM mode can be generated at the output port.

The structure of the DC-PCF designed in this paper is shown in Figure 1, where d=1.2 μm, Λ=2.4 μm, and a perfectly matched layer with a thickness of 3 µm is added outside the simulation area. Six air holes were removed on the left side of the fiber, and the air holes in the middle were left, thus forming the ring fiber core with few-mode on the left side. One air hole was removed at a position 4Λ away from the center of the FMFC on the right, and six air holes around it were filled with TS-RIMS. According to the design principle of the photonic crystal fiber, when the duty cycle is d/Λ=0.5 and λ/Λ≥0.34, it can be guaranteed to transmit the fundamental mode [36]. Two air holes are left between the cores to reduce the impact after filling the TS-RIMS around the SMFC. This can effectively prevent the filled liquid from affecting the effective refractive index of the mode in the FMFC. The TS-RIMS used in this paper was produced by Cargille Laboratories Inc. The dispersion properties of this material conform to the Cauchy model [37]. At 25 °C, its refractive index at a wavelength of 1550 nm is 1.3043, and its thermo-optical coefficient is −0.000335 refractive index units per degree Celsius (RIU/°C) in the range of 15–35 °C. In order to facilitate the splicing with the traditional silica fiber, the background material of the fiber uses silica, and its dispersion model satisfies the Sellmeier equation [38]. The refractive index of silica is 1.444 when the wavelength is 1550 nm. The thermo-optical coefficient of silica is $8.5\times 10^{-6}$ RIU/°C. This indicates that the refractive index of the liquid can be adjusted by changing the external temperature.

**Figure 2:**
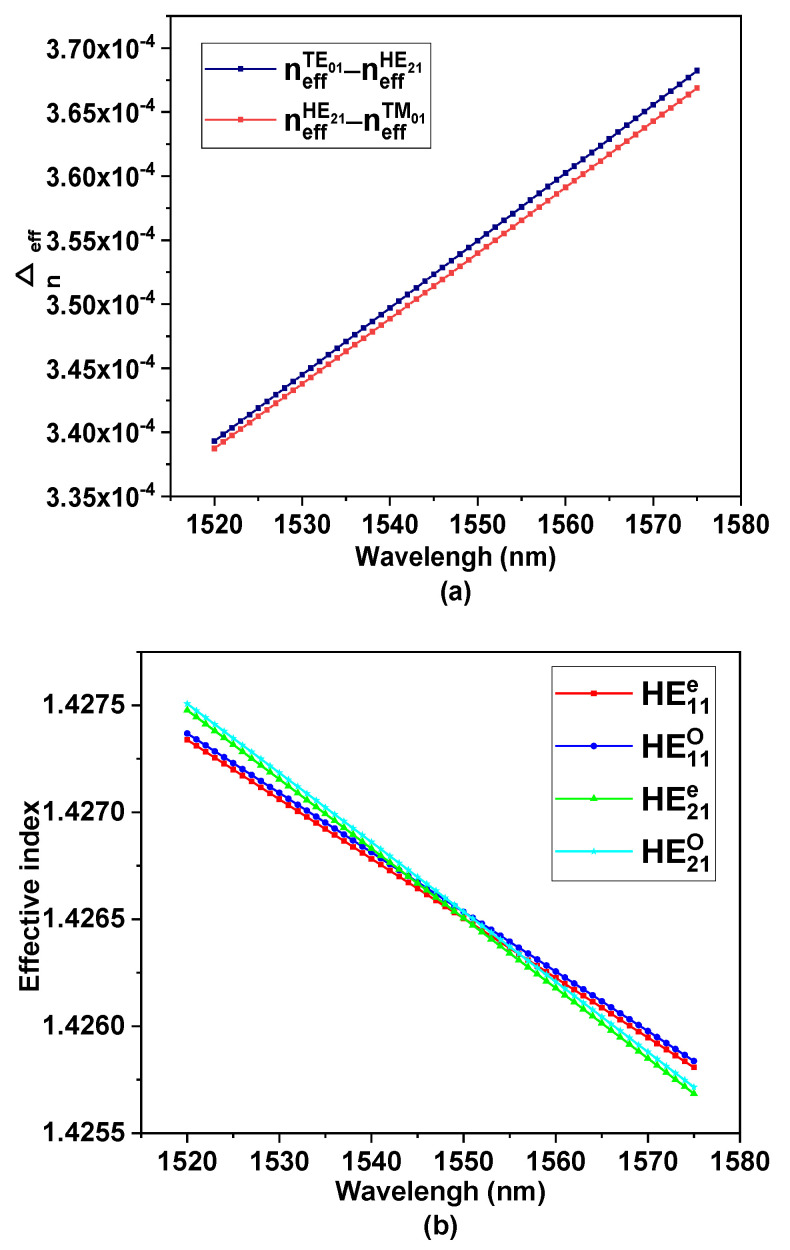
(**a**) ERI differences between different vector modes in few-mode core. (**b**) Dispersion curves of the odd mode and even mode of HE_11_ in the SMFC and HE_21_ in the FMFC.

In the DC-PCF design process, the effective refractive index (ERI) of the HE_11_ mode in the SMFC is close to that of the HE_21_ mode in the FMFC by adjusting the size of the structure. Figure 2a shows the ERI difference (ERID) of the TE_01_/TM_01_ and HE_21_ modes in the FMFC. It can be seen that the minimum ERI difference between 1520–1575 nm is $\Delta n_{eff}>1.8\times 10^{-4}$, which can effectively avoid the degeneracy of these modes [39]. By adjusting the structural parameters, the ERI of the HE_11_ mode in the SMFC is close to the HE_21_ mode in the FMFC. As shown in Figure 2b, the dispersion curves of the HE_11_ mode in the SMFC and the HE_21_ mode in the FMFC are calculated. As can be seen from the figure, this structural parameter can make the intersection of the ERI of the two modes appear at a wavelength of 1550 nm. Since the six air holes around the SMFC are filled with TS-RIMS, the ERI of the fundamental mode of the SMFC needs to be smaller than the high-order mode of the FMFC through the structural design before filling the TS-RIMS. After filling the TS-RIMS, the ERI of the fundamental mode of the SMFC will be increased, which is close to the ERI of the HE_21_ mode of the FMFC.

## 4. Results and Properties

**Figure 3:**
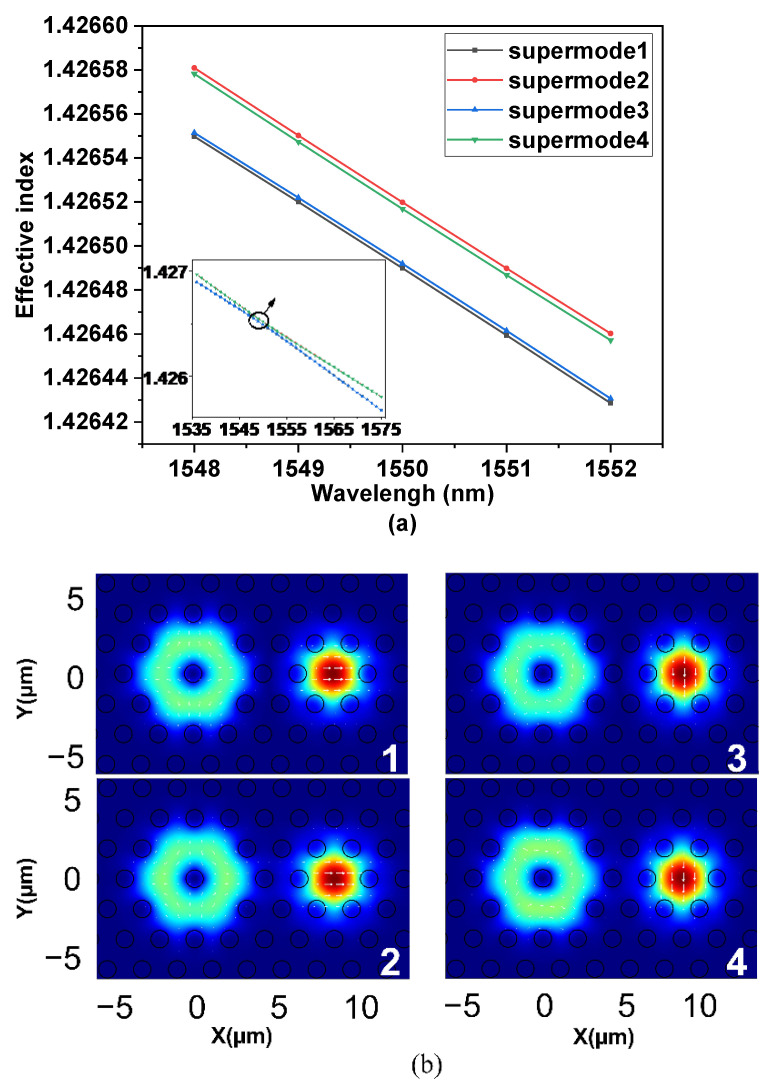
(**a**) Dispersion curves of the four supermodes created by the resonance between HE_11_ and HE_21_ modes. (**b**) Modal energy distributions of the four supermodes created by the resonance between HE_11_ and HE_21_ modes.

In this paper, the full vector finite element method was used to analyze the coupling efficiency, coupling length, purity and bandwidth of each OAM mode of the generator. Firstly, the DC-PCF was modeled in COMSOL, then the dispersion characteristics of the supermodes were analyzed and the phase matching point was found between the HE_11_ and HE_21_ modes supported by the SMFC and the FMFC, respectively. The dispersion curve of the odd and even modes of HE_11_ mode in the SMFC and HE_21_ mode in the FMFC in DC-PCF is depicted in the inset of Figure 3a. Figure 3a shows the dispersion curve of the supermodes near the resonance wavelength. Figure 3b is the energy distribution of the supermodes at the resonance wavelength. According to PSCC, the characteristics of the generator when inputting light with different polarization states were analyzed through the superposition between supermodes. According to Equation (2), the overall mode field distribution and phase distribution were analyzed by the superposition supermodes. Next, the energy conversion efficiency (ECE) of the OAM mode generator was analyzed. Moreover, the purity of the OAM mode was calculated based on the Fourier series expansion method [40]. In [40] it’s the purity is assessed by calculating the energy proportion of the OAM mode, which can be expressed as Equation (9).

(9) $$Q_{l}=\frac{P_{l}}{\sum_{n}{}_{-\infty }^{+\infty }P_{n}}$$

where $\sum_{n}{}_{-\infty }^{+\infty }P_{n}=1$, $P_{l}={\int_{0}^{\infty }\left|a_{l}(\rho ,z)\right|}^{2}\rho d\rho$ is the normalized energy of the OAM mode with a topological charge of *l*, $a_{l}=1/\sqrt{2\pi }\int_{0}^{2\pi }u(\rho ,\varphi ,z)\exp(-il\varphi )d\varphi$, u(ρ,φ,z) is any field distribution in the paraxial state of light propagation, and by using Fourier series expansion, one can obtain $u(\rho ,\varphi ,z)=1/\sqrt{2\pi }\sum {}_{-\infty }^{+\infty }\sum_{l=-\infty }^{l=+\infty }a_{n}(\rho ,z)\exp(il\varphi )$.

According to theoretical analysis, it can be concluded that when CPFM or LPFM is input, the OAM mode can be efficiently generated when the transmission distance *L* satisfies the Equations (4) and (7) respectively. According to Equations (4) and (7), it is necessary to obtain the propagation constants of the four supermodes produced by the resonance of the HE_11_ mode in the SMFC and HE_21_ mode in the FMFC. Next, we need to find the coefficients of the PSCC ($2k_{1}$,$2k_{2}+1$,$2k_{3}$,$2k_{4}+1$) when the CPFM is injected. and the coefficients of the PSCC ($2q_{1}$,$2q_{2}+1$,$2q_{3}+1/2$,$2q_{4}+3/2$) with LPFM input. After calculation, when the CPFM is input, the mantissas of the coefficients of the PSCC when *L* is roughly equal to 28 mm are 7.96, 9.08, 8.04 and 8.97, respectively. When LPFM is input, the mantissas of PSCC are 5.99, 1.00, 2.50 and 3.49 when *L* is approximately equal to 650 mm.

### 4.1. Characteristics of OAM Generator with LPFM Input

**Figure 4:**
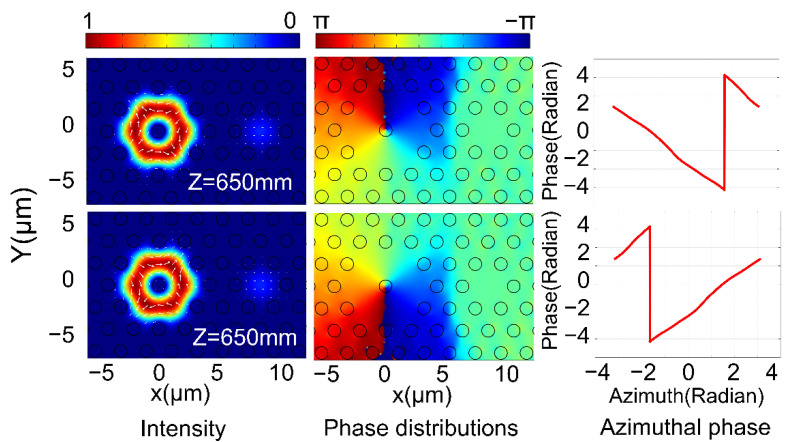
The intensity distribution, phase distribution and the angular phase change in the FMFC for the generated ${\mathrm{OAM}}_{\pm 1}^{\pm }$ modes in FMFC at 1550 nm when the LPFM is input. The top image shows the ${\mathrm{OAM}}_{+1}^{+}$ mode, and the bottom image depicts the ${\mathrm{OAM}}_{-1}^{-}$ mode.

According to Equation (2), the overall mode field distribution and phase distribution were analyzed by the superposition supermodes when LPFM is input. Figure 4 shows the mode field distribution and phase distribution in the DC-PCF, and the angular phase distribution in the FMFC when the transmission distance is 650 mm.

**Figure 5:**
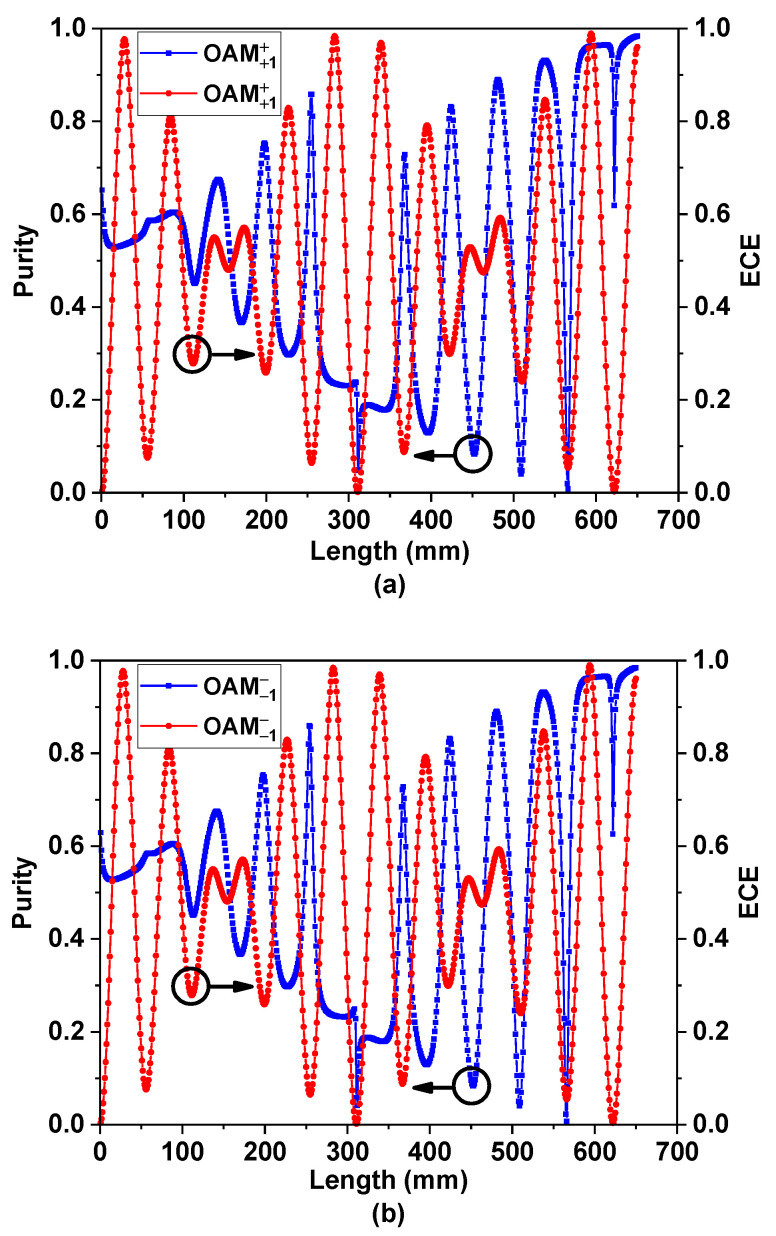
(**a**) Purity and energy conversion efficiency (ECE) of +1 order OAM mode with LPFM input. (**b**) Purity and energy conversion efficiency (ECE) of -1 order OAM mode with LPFM input.

Figure 5a,b are the variation curves of the purity and ECE of the ±1 order OAM mode with the change of *L*. It can be seen that its purity gradually increases, and the coupling efficiency changes periodically. However, when the coupling distance *L* = 650 mm, the conversion efficiency and purity of the ±1 order OAM mode are both higher: the +1 order OAM mode is 96.04% and 98.39%, and the −1 order OAM mode is 96.04% and 98.39%, respectively.

When the LPFM is input, its energy changes periodically between the two cores, which conforms to the coupled mode theory. The purity of the generated OAM mode changes irregularly because the phase difference between the supermodes changes with the increase in the transmission distance. According to the PSCC, it was calculated that the device can efficiently produce high-purity OAM mode when the coupling distance is about 650 mm, and the simulation results are consistent with this. These results show that the device can excite the ${\mathrm{OAM}}_{\pm 1}^{\pm }$ mode when the LPFM is input.

### 4.2. Characteristics of OAM Generator with CPFM Input

**Figure 6:**
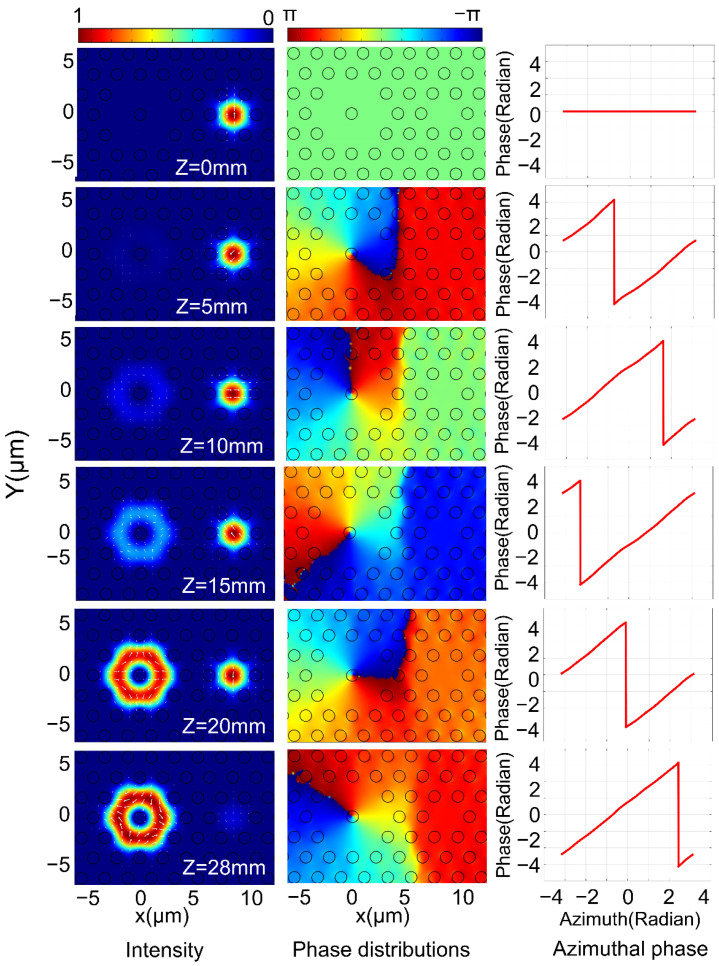
The intensity profiles, phase distributions and azimuthal phase variations for the generated ${\mathrm{OAM}}_{+1}^{+}$ mode in FMFC at 1550 nm with Z change from 0 mm to 28 mm when the CPFM is input.

**Figure 7:**
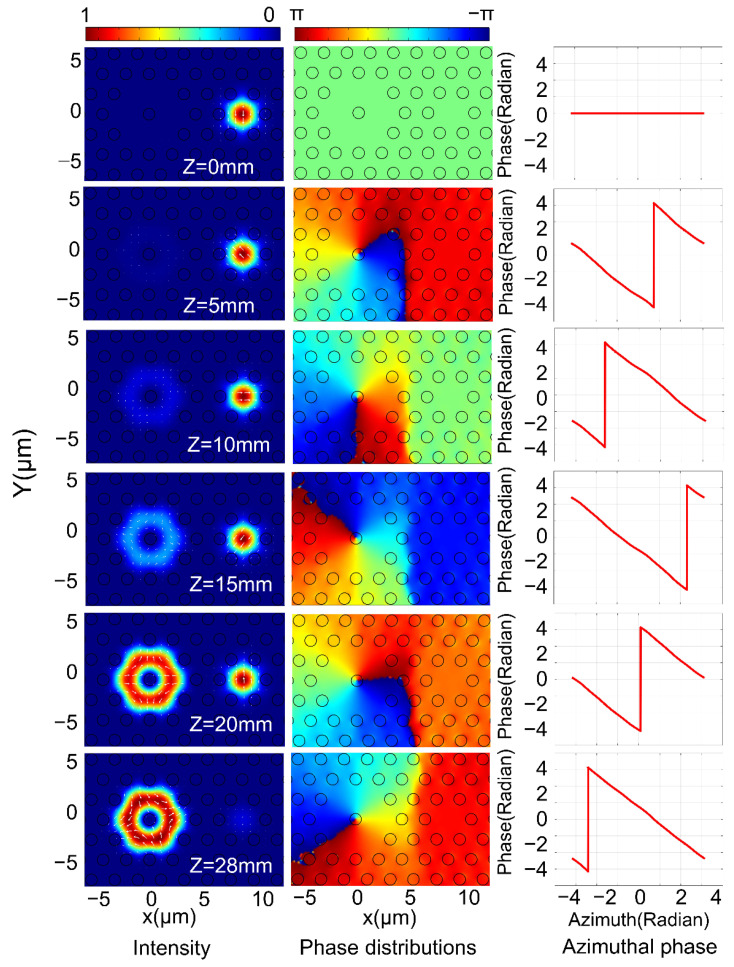
The intensity distribution, phase distribution and the angular phase change in the FMFC for the generated ${\mathrm{OAM}}_{-1}^{-}$ mode in FMFC at 1550 nm with Z change from 0 mm to 28 mm when the CPFM is input.

Figure 6 and Figure 7 show the mode field change, phase distribution and angular phase distribution in the FMFC with the transmission distance change from 0 mm to 28 mm.

**Figure 8:**
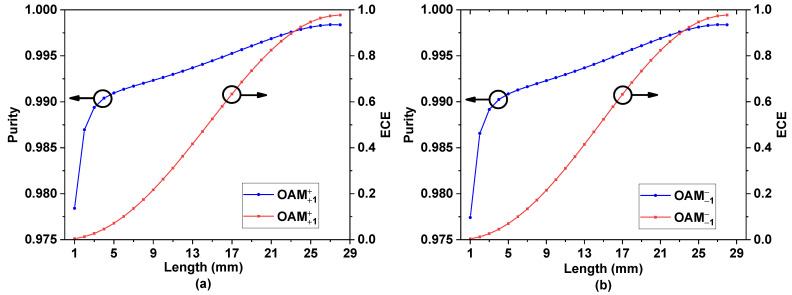
(**a**) Purity and energy conversion efficiency (ECE) of +1 order OAM mode with CPFM input. (**b**) Purity and energy conversion efficiency (ECE) of −1 order OAM mode with CPFM input.

Figure 8a,b are the variation curves of the purity and conversion efficiency of the ±1 order OAM mode with the change of *L* when the CPFM is incident. When the coupling distance *L* = 28 mm, the ECE and purity of the ±1 order OAM mode reach their highest: the +1 order OAM mode is 97.72% and 99.83%, and the −1 order OAM mode is 97.71% and 99.83%, respectively.

According to the simulation results, when the CPFM is input, the energy change conforms to the coupled mode theory. However, the purity of the generated OAM mode remains at a high value because the phase difference between the odd mode and the even mode of the input CPFM itself is π/2. According to Figure 6 and Figure 7, it can be seen that the coupling efficiency and purity reach their highest when the coupling distance is 28 mm, which is almost identical to the results calculated based on the PSCC. In summary, it can be shown that the CPFM input requires a shorter coupling distance to produce modes. It can be seen from Figure 4, Figure 5, Figure 6, Figure 7 and Figure 8 that the OAM mode with a topological charge of m = ±1 can be excited with LPFM and CPFM input.

### 4.3. Thermal Tuning Characteristics of OAM Mode Generator

**Figure 9:**
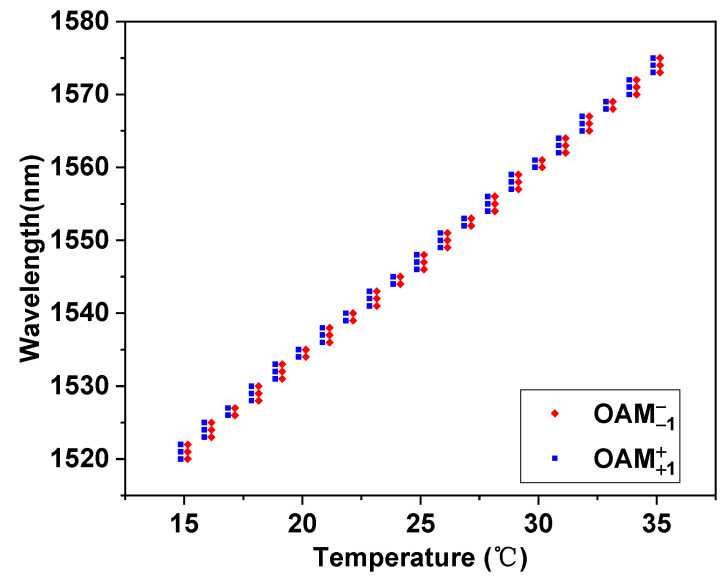
The variations in coupling wavelengths as temperature changes for different modes.

The above analysis was carried out at a wavelength of 1550 nm. Next, we will discuss the bandwidth characteristics of the OAM mode generator of the fiber. Because the TS-RIMS selected in this article has a thermo-optical coefficient of −0.000335 refractive index units per degree Celsius (RIU/°C) within the range of 15 °C and 35 °C; therefore, the refractive index of the filling liquid can be controlled by changing the temperature, and then the phase matching condition of the fundamental mode in the SMFC and the higher-order mode in the FMFC can be tuned to make the OAM mode generator work at different wavelengths. Since the length of the DC-PCF is only 28 mm when circularly polarized light is input, it is not only simple to manufacture, but the performance is also stable. Therefore, the bandwidth of the OAM mode generator is analyzed only when the CPFM is input. Figure 9 depicts the variations in coupling wavelengths as the temperature changes for different modes.

**Figure 10:**
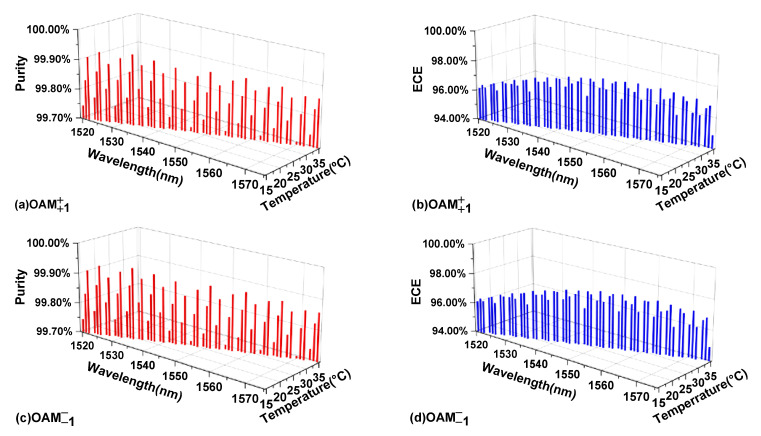
(**a**,**b**) The purity and energy conversion efficiency (ECE) of the +1st order OAM mode in the wavelength range of 1520–1575 nm when CPFM is input. (**c**,**d**) The purity and energy conversion efficiency (ECE) of the −1st order OAM mode in the wavelength range of 1520–1575 nm when CPFM is input.

Figure 10 shows the purity and ECE of the ±1 order OAM mode in the wavelength range of 1520 nm–1575 nm when CPFM is input. It can be concluded that the purity of the ±1 order OAM mode produced by the OAM mode generator without changing its structure is above 99%, and the ECE is more than 95%.

The variation in wavelength will change the propagation constant of each supermode, and the corresponding phase matching coefficient will, in turn, affect its coupling state. As the wavelength deviates from the resonance point, the purity and coupling efficiency of the OAM mode are reduced, and the phase distribution of the wavefront is no longer a spiral wavefront. In order to increase its bandwidth, the mode in the two cores can meet the phase matching condition again through temperature adjustment, without changing the structure. We analyzed the wavefront phase distribution and energy coupling process at different wavelengths, and their states are similar to those shown in Figure 6 and Figure 7. The results show that the designed device can efficiently produce high-purity OAM modes at each operating wavelength.

The proposed OAM mode generator based on the DC-PCF is flexible and adjustable. By filling the six air holes around the SMFC with any functional material with an adjustable refractive index, the resonance conditions of the core can be changed. In addition to the TS-RIMS selected in this article, there are also magnetically regulated magnetic fluids [41] and temperature-adjusted liquid crystals [42], etc.

## 5. Discussion of Fabrication Issues

The proposed DC-PCF selectively filled with liquid can be fabricated in principle. We will discuss the fabrication issues of the designed PCF, although our current work is focused on the fiber design and theoretical aspects.

At present, the following two main methods are used to selectively fill PCF with special shapes. First, the UV-curing adhesive is applied to the surface of the conical optical fiber needle. By controlling the optical fiber needle, the UV-curing adhesive on the surface is applied to the air holes that do not need to be filled with liquid to block them, leaving the air holes that need to be filled with liquid. After the UV-curing adhesive is solidified, the blocked end of the PCF is immersed in the liquid, and the liquid fills the air holes by capillary action. This method has a high degree of freedom and a wide range of application. It is the most widely used filling method at present [43]. Secondly, the shape of the region to be filled is etched on the side of PCF by the high-energy targeting feature of a femtosecond laser. Subsequently, the PCF end face at the etched end is welded and collapsed, so that the liquid can only enter the PCF through the etched area. Finally, the etched and welded part can be cut off [44].

The six air holes around the single-mode core of the DC-PCF are filled, this selective filling can be achieved by the above methods.

## 6. Conclusions

In this paper, a thermally tunable OAM generator has been proposed. Firstly, the influence of the polarization state of the input light on its characteristics was analyzed by the full-vector finite element method. According to the results, the ±1 order OAM mode can be excited by inputting CPFM or LPFM, and their required coupling lengths are 28 mm and 650 mm, respectively. Then, the phase matching points of HE_11_ in SMFC and HE_21_ in FMFC were tuned by controlling the temperature to achieve the change in the resonance wavelength. The results show that the device can effectively excite a high-purity OAM mode with wavelengths ranging from 1520 nm to 1575 nm by adjusting the temperature between 15 °C and 35 °C. The purity of the ±1 order OAM mode produced by the OAM mode generator surpassed 99%, and the ECE was above 95%. Therefore, the proposed design can be used efficiently in all-fiber OAM mode multiplexing communication systems.

## Data Availability

Data sharing not applicable.
